# Novel approaches for the lipid sponge phase crystallization of the *Rhodobacter sphaeroides* photosynthetic reaction center

**DOI:** 10.1107/S2052252520012142

**Published:** 2020-10-03

**Authors:** Georgii Selikhanov, Tatiana Fufina, Lyudmila Vasilieva, Christian Betzel, Azat Gabdulkhakov

**Affiliations:** a Institute of Protein Research, Russian Academy of Sciences, Institutskaya 4, Puschino, Moscow region 142290, Russian Federation; b Institute of Basic Biological Problems, Russian Academy of Sciences, Institutskaya 2, Puschino, Moscow region 142290, Russian Federation; c Institute of Biochemistry and Molecular Biology, University of Hamburg, at Deutsches Elektronen-Synchrotron (DESY), Notkestrasse 85, Hamburg, 22607, Germany; d The Hamburg Centre for Ultrafast Imaging (CUI), Luruper Chaussee 149, Hamburg, 22761, Germany

**Keywords:** mesophase crystallization, lipid sponge phase, monoolein, photosynthetic reaction centers, *Rhodobacter sphaeroides*, serial crystallography

## Abstract

With the recent developments in the field of free-electron-laser-based serial femtosecond crystallography, the necessity to obtain a large number of high-quality crystals has emerged. In this work crystallization techniques were selected, tested and optimized for the lipid mesophase crystallization of the *Rhodobacter sphaeroides* membrane pigment-protein complex, known as the photosynthetic reaction center (RC). Novel approaches for lipid sponge phase crystallization in comparatively large volumes using Hamilton gas-tight glass syringes and plastic pipetting tips are described. An analysis of RC crystal structures obtained by lipid mesophase crystallization revealed non-native ligands that displaced the native electron-transfer cofactors (carotenoid sphero­idene and a ubi­quinone molecule) from their binding pockets. These ligands were identified and were found to be lipids that are major mesophase components. The selection of distinct co-crystallization conditions with the missing cofactors facilitated the restoration of sphero­idene in its binding site.

## Abstract 2

Novel approaches for lipid sponge phase crystallization in comparatively large volumes using Hamilton gas-tight glass syringes and plastic pipetting tips are described. The selection of distinct co-crystallization conditions allowed to restore one of the natural ligands displaced by monoolein to its binding site.

## 1. Introduction   

Although water-soluble proteins can be crystallized and studied by X-ray structural analysis with relative success, membrane proteins remain problematic objects for analysis of their spatial structure. Crystallization of membrane proteins using detergents is often ineffective, since large micelles form around the proteins that prevent the formation of crystalline contacts (Bill *et al.*, 2011 ▸). One of techniques developed for membrane protein crystallization is crystallization using lipid mesophases. In 1996, crystallization in the lipid cubic phase (LCP) was first used and the structure of bacteriorhodopsin was successfully determined (Landau & Rosenbusch, 1996 ▸). Later on its basis lipid sponge phase (LSP) crystallization method was introduced (Wadsten *et al.*, 2006 ▸). In both cases, matrix lipids form an environment that mimics the cell membrane, which contributes to the crystallization process. Lipid mesophase crystallization has some advantages over crystallization by the detergent-based vapor-diffusion technique. Crystals obtained by this method often have less solvent in their overall composition, higher symmetry, increased durability and, most importantly, lipid mesophase crystallization is often the only effective crystallization method for some proteins. In recent years, the proportion of new structures obtained by applying this technique has increased compared with detergent-based crystallization (Ishchenko, Abola *et al.*, 2017 ▸).

Serial crystallography, especially time-resolved experiments on X-ray free-electron laser (XFEL) equipment, requires a significant number of small crystals, and crystallization in lipid mesophase has also proven to be a useful tool for sample preparation. The principle of the ‘diffraction before destruction’ method requires a special approach to deliver crystals to the X-ray beam, and viscous lipid carriers provide certain advantages in this regard. The LCP jet delivery system that is often used for viscous crystal carrier matrices has lower sample consumption compared with a liquid jet. To take advantage of low consumption it is possible to mix the crystal solution with a wide variety of viscous matrices with broad hydro­phobic and hydro­philic compatibility (Nam, 2019 ▸). However, this additional mixing step could introduce damage and reduce the diffraction quality of the crystals, and therefore it is more convenient to grow the crystals in viscous media such as LCP or LSP at the beginning.

**Figure 1:**
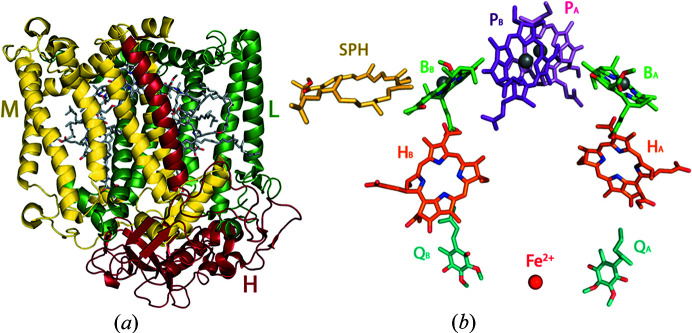
(*a*) Structure of the *Rba. sphaeroides* RC (PDB entry 3v3y). The L, M and H subunits are shown in green, yellow and red, respectively; (*b*) RC cofactors organized in two electron-transfer chains. P_A_ and P_B_, bacterio­chloro­phyll dimer; B_A_ and B_B_, bacterio­chloro­phyll monomers; H_A_ and H_B_, bacteriopheophytins; Q_A_, primary ubi­quinone Q_B_, secondary ubi­quinone; SPH, sphero­idene.

In this work, the photosynthetic reaction center of the purple bacteria *Rhodobacter (Rba.) sphaeroides* was crystallized using various crystallization techniques. This transmembrane pigment-protein complex consists of three subunits L, M and H, and ten electron-transfer cofactors, including bacterio­chloro­phylls, bacteriopheophytins, ubi­quinones, non-heme iron ions and carotenoid sphero­idene (Fig. 1 ▸) (Feher *et al.*, 1989 ▸). We used *in meso* crystallization in the LCP and LSP, as well as the detergent-based vapor-diffusion method (*in surfo*). The purpose of this work was to select the crystallization conditions and develop an efficient technique for obtaining a large number of crystals in the LSP for further use in serial crystallography. Structural data presented here were obtained via conventional rotation crystallography for rapid quality checking of the crystals obtained.

## 2. Results   

### 2.1. Crystalliation of the photosynthetic RC and analysis of the structures obtained   

**Figure 2:**
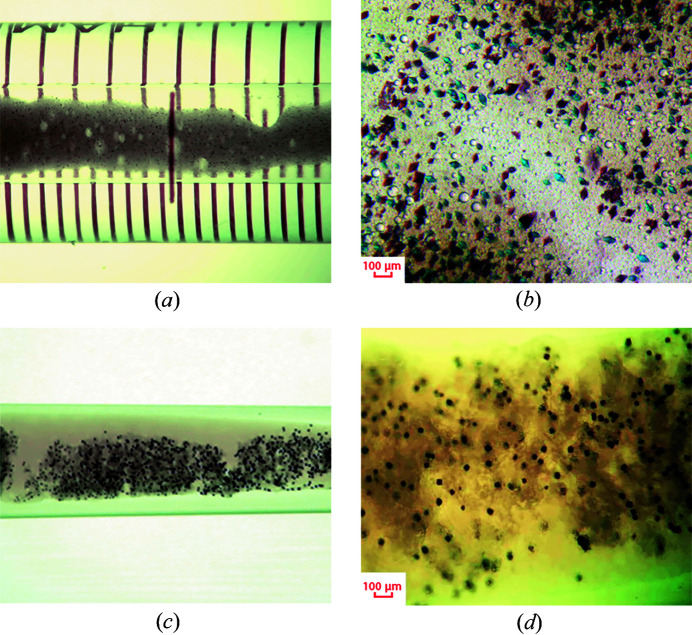
RC crystals obtained in LSP. (*a*) Inside a Hamilton gas-tight glass syringe; (*b*) a Hamilton gas-tight glass syringe under magnification; (*c*) inside a sealed plastic tip; (*d*) a sealed plastic tip under magnification.

Initially, for RC crystallization we used previously described methods of mesophase crystallization in the LCP (Katona *et al.*, 2003 ▸) and in the LSP (Wadsten *et al.*, 2006 ▸) with slightly modified conditions. For LSP crystallization we used the hanging-drop vapor-diffusion method and for LCP crystallization a microbatch crystallization approach. High-quality protein crystals for X-ray diffraction were obtained. Diffraction data from single crystals at 100 K were collected at the ESRF, Grenoble; spatial crystal structures with a maximum resolution of 2.1 Å were resolved and refined. Furthermore, we developed new techniques for the crystallization of reaction centers in the LSP in Hamilton gas-tight glass syringes and in plastic pipetting tips to increase the number of crystals obtained (Fig. 2 ▸). These techniques are intended to be used to obtain a sufficient number of crystals for serial crystallography experiments.

In order to have a ‘control and reference’ crystal structure without lipids we carried out RC crystallization by applying the detergent-based hanging-drop vapor-diffusion method utilizing conditions we used in earlier work (Gabdulkhakov *et al.*, 2013 ▸). The resulting crystals were of sufficient quality to obtain a 2.1 Å crystal structure. This *in surfo* structure was used for comparison with *in meso* structures.

It should be noted that the RC of *Rba. sphaeroides* is one of the most well studied membrane complexes. The number of cofactors in this RC was first determined by spectral methods during the study of the electron-transfer process and later confirmed by X-ray crystal structure analysis (Feher *et al.*, 1989 ▸).

Analysis of spatial structures derived from RC crystals grown in the presence of lipids showed two significant differences compared with RC structures obtained by *in surfo* vapor-diffusion crystallization.

**Figure 3:**
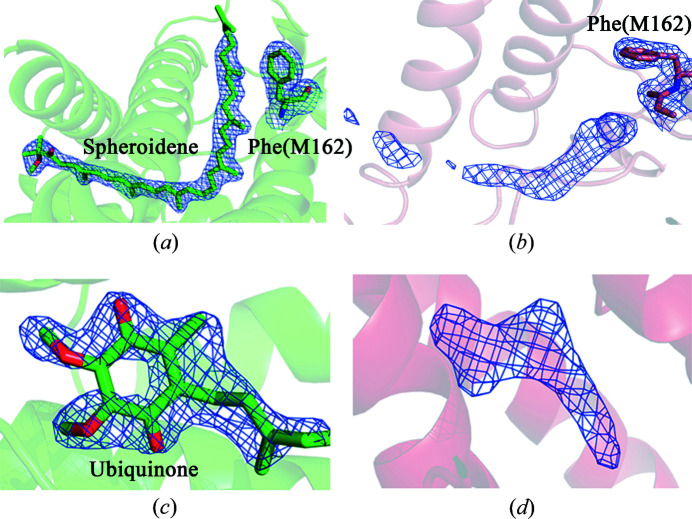
Fragment of the electron density map of the *Rba. sphaeroides* RC structure. (*a*) In the carotenoid binding site, under *in surfo* conditions, at 2.1 Å resolution, 1.6σ; (*b*) in the carotenoid binding site, in the LSP, at 2.1 Å resolution, 1.0σ; (*c*) in the *Q*_B_ binding site, under *in surfo* conditions, at 2.1 Å resolution, 1.6σ; (*d*) in the *Q*_B_ binding site, in the LSP, at 2.1 Å resolution, 1.0σ.

The first difference is the absence of carotenoid sphero­idene in structures derived from lipid mesophase crystals [Figs. 3 ▸(*a*) and 3 ▸(*b*)]. In the carotenoid-binding region an electron density is present that resembles a carotenoid molecule. However, the size and shape of this electron density do not allow the fitting of a full-sized sphero­idene molecule into it. In addition, there is a clear visible change in the protein itself. The M162 phenyl­alanine side group forms a kind of ‘closed’ conformation. In this position, the M162Phe side group occupies space that in the previously described RC structures is occupied by a sphero­idene molecule.

The second difference is the presence of electron density in the secondary ubi­quinone *Q*_B_ pocket, which does not correspond to a ubi­quinone molecule [Figs. 3 ▸(*c*) and 3 ▸(*d*)]. In some *in surfo* RC structures this region has weak electron density, which is apparently caused by the mobility of this ubi­quinone. However, there are structures in which the electron density for *Q*_B_ is strong and clearly visible like in our ‘control’ crystal structure obtained without lipids. In the *in meso* RC structures, a different picture is observed. There is a distinct electron density in the *Q*_B_ pocket which looks unlike an ubi­quinone molecule. We assume that another molecule occupies the *Q*_B_ pocket, displacing the natural ubi­quinone.

### 2.2. Spectral analysis of dissolved crystals   

**Figure 4:**
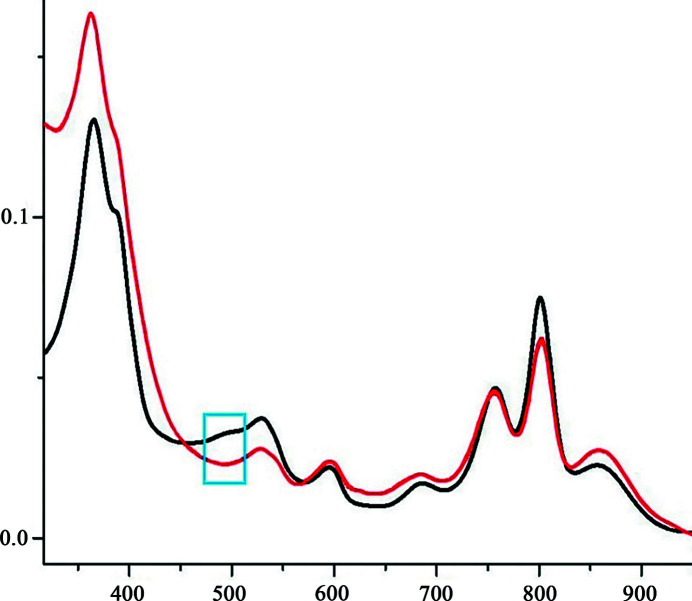
Absorption spectra of the *Rba. sphaeroides* RC solutions obtained by dissolution of *in surfo* crystals (black curve) and *in meso* crystals (red curve). The highlighted area corresponds to the carotenoid absorption region.

The presence of the carotenoid molecule in the RC can be determined by the absorption spectrum in the region of this pigment-protein complex near 490 nm. To confirm the absence of carotenoid in the RC crystals obtained by lipid mesophase crystallization, we dissolved *in meso* crystals and *in surfo* crystals in detergent-containing buffer. For that purpose we used crystals from the same crystallization trials from which we took samples for X-ray structural analysis. Absorption spectra of dissolved pigment-protein complexes were measured and compared (Fig. 4 ▸). It can be seen that there is a small absorption band in the region of 490 nm in the spectrum of the *in surfo* RC, characteristic of a sphero­idene molecule, which is not observed in the spectrum of *in meso* RC, and which is in agreement with the absence of this molecule in the *in meso* RCs.

### 2.3. Determination of non-native ligands   

In an attempt to identify the molecules replacing the native cofactors in their binding pockets we discovered that the observed electron densities could be described well by monoolein molecules. Monoolein is the most commonly used matrix lipid for mesophase crystallization. It is a representative of mono­acyl­glycerols (MAGs).

**Figure 5:**
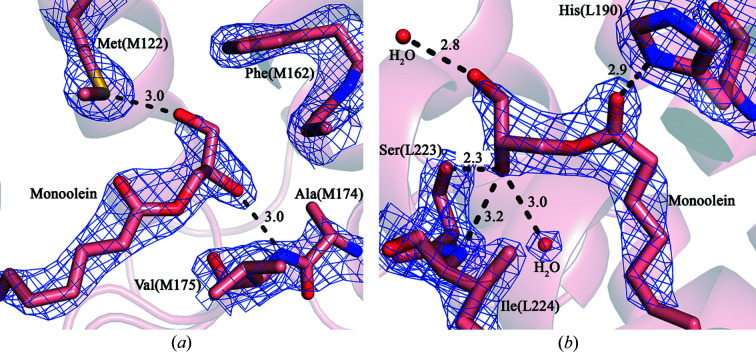
Monoolein molecules fitted into an electron density of interest in the *Rba. sphaeroides* RC (2.1 Å resolution, 1.6σ). (*a*) Carotenoid binding site and (*b*) *Q*_B_ binding site.

Apparently, owing to good steric compliance, monoolein molecules are able to penetrate the binding sites of sphero­idene and secondary ubi­quinone in the RC, thus replacing the native cofactors [Figs. 5 ▸(*a*) and 5 ▸(*b*)].

### 2.4. Co-crystallization with carotenoid and ubiquinone   

To solve the problem of non-native ligands, we attempted to increase the concentration of the missing cofactors in order to competitively displace the non-native ligands. It was shown that to increase the occupancy of *Q*_B_ in RC crystals, ubi­quinone could be added to the mother liquor as described in the work by Axelrod *et al.* (2000 ▸). In these experiments the soaking method proved to be effective in the case of *in surfo* crystals. Another widespread crystallographic approach is a co-crystallization whereby the substance is added to the crystallization mix during its preparation (Müller, 2017 ▸).

We attempted to both co-crystallize reaction centers in the LSP with the missing cofactors and soak mature *in meso* crystals in cofactor-containing solution. Co-crystallization and soaking were performed with the addition of sphero­idene and ubi­quinone solutions.

First, we tried soaking mature crystals. We added sphero­idene dissolved in acetone and ubi­quinone dissolved in ethyl acetate to the hanging drops that contained LSP with embedded RC crystals obtained during the vapor diffusion experiments. We tried different volumes of additives and, in the most extreme cases, we completely covered LSP with the organic solvent. We proposed that water channels that exist in LSP as well as in LCP would facilitate the delivery of cofactors to the crystals (Briggs *et al.*, 1996 ▸; Engström *et al.*, 1998 ▸). After three weeks of soaking, these crystals were used for diffraction data collection.

In the case of soaking with ubi­quinone the samples showed no differences in their structure in the *Q*_B_ binding site compared with the control *in meso* structure. Therefore, the ubi­quinone soaking method proved to be ineffective in the case of *in meso* crystallization.

For sphero­idene soaking we had different results for every crystal. In some cases we saw no differences in the carotenoid binding site, and in others carotenoid completely displaced the non-native ligand. Many structures had hybrid densities that confirmed only partial displacement of the monoolein. The reason for such results may be the uneven penetration of the sphero­idene solution into different parts of the LSP.

The first problem with the soaking method is that it seems to be difficult to apply on a larger scale due to uneven solvent penetration. Another problem is that most crystals are damaged by the organic solvents (both acetone and ethyl acetate) and their diffraction quality noticeably decreases.

**Figure 6:**
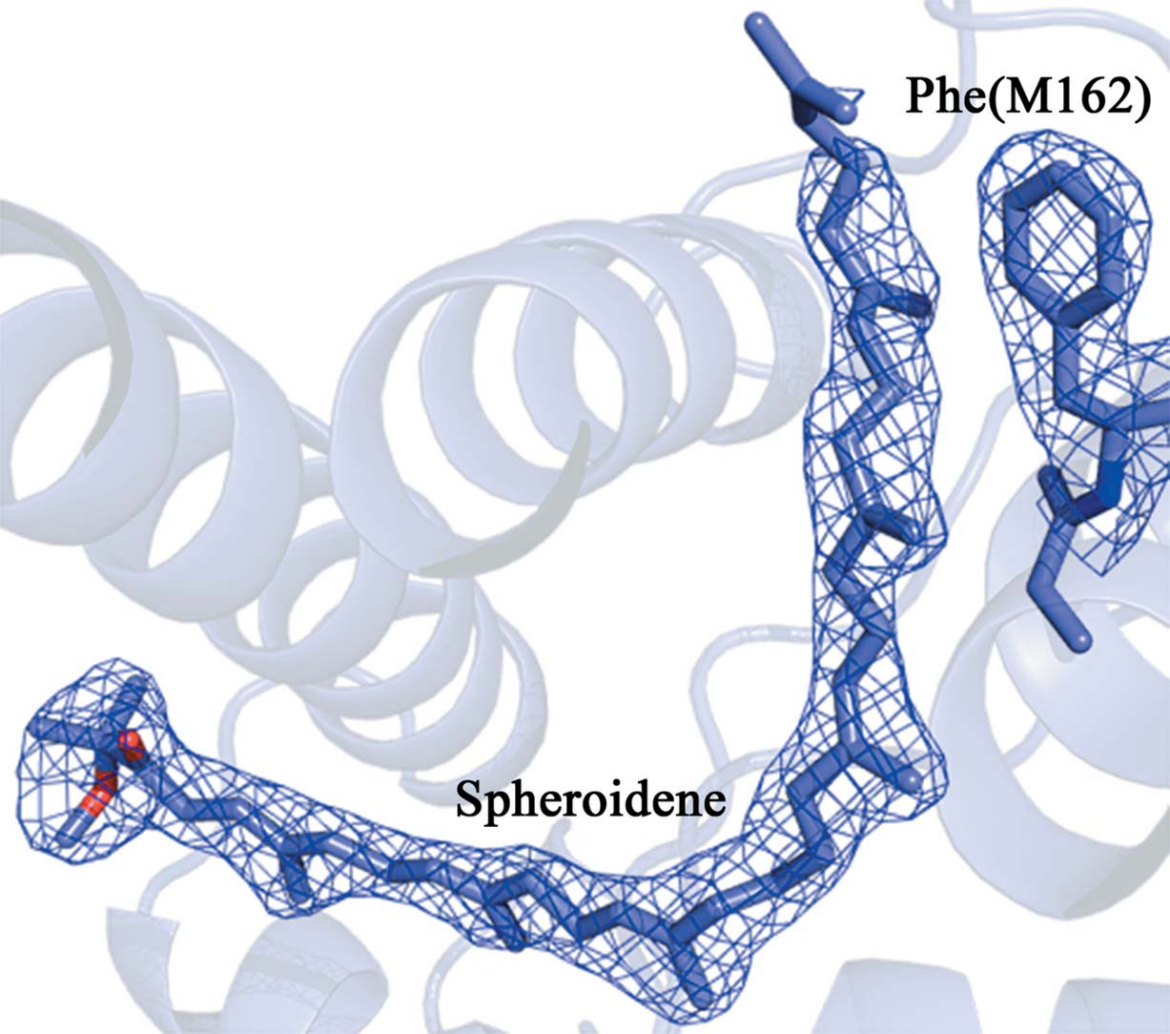
Fragment of the electron density map of the *Rba. sphaeroides* RC structure in the carotenoid binding site. The sample co-crystallized with carotenoid in the LSP (2.1 Å resolution, 1.6 σ).

Later co-crystallization experiments were performed. We added carotenoid solution to the crystallization mix. By titration it was shown that when the final concentration of acetone is approximately 5% or more the quality of the resulting crystals deteriorates, or they even stop appearing. At a final acetone concentration of 2.5%, no negative effects on the crystallization or crystal quality were observed. By adding an acetone solution of sphero­idene to the final acetone concentration of 2.5%, crystals were obtained in which the sphero­idene completely displaced monoolein from its binding site (Fig. 6 ▸).

A series of co-crystallization experiments were carried out with the addition of various volumes of ubi­quinone solution. Even at high solvent concentration, when the crystal quality deteriorated significantly, the *Q*_B_ binding site remained occupied by monoolein. The reason for this may be the high affinity of monoolein for this binding site and/or too much monoolein in the crystallization mixture with respect to ubi­quinone. Another possible reason is the relatively low affinity of ubi­quinone to the *Q*_B_ binding site associated with its function: ubi­quinone comes and goes easily.

### 2.5. Increasing the number of crystals: syringe-based and in-nozzle crystallization   

To increase the number of crystals for further use in serial crystallography, it is necessary to enlarge the volume of crystallization mixtures.

Crystallization using lipid mesophases has a characteristic feature. The crystallization process strongly depends on the ratio of the area of the water/lipid phase interface and the volume of the crystallization mixture. For this reason, attempts to crystallize RC in LSP by vapor diffusion in a sitting drop failed.

In order to increase the total volume of the crystallization mixture while maintaining a sufficient phase boundary surface, reaction centers were crystallized in Hamilton gas-tight glass syringes and later in plastic tips. Both methods allowed us to obtain sample quantities suitable for serial crystallography studies. In the case of syringe crystallization the best resolution was only 2.8 Å. Applying the in-tips technique allowed us to obtain a resolution up to 1.8 Å. Syringe-based and in-tip crystallization techniques are described in detail in the Methods section[Sec sec4].

## 3. Discussion   

In this work novel crystallization techniques for lipid mesophase crystallization of the *Rba. sphaeroides* RC were tested and optimized, and we obtained a considerable number of high-quality crystals. This successful approach can be further used to produce samples for serial crystallography experiments. We also showed that, despite the many advantages of lipid mesophase crystallization, it has some disadvantages. We found displacements of two native cofactors by non-native ligands in the *in meso* structures together with changes in the spatial arrangement of some amino acid residues.

Analysis of RC structures of some purple bacteria that are currently available in the Protein Data Bank identified other cases where crystallization in the lipid mesophase led to the replacement of natural cofactors with geometrically similar molecules. The binding sites of the ubi­quinone *Q*_B_ and the carotenoid sphero­idene in the *in meso* crystal structures deposited by other authors also contain electron densities that do not correspond to the native cofactors (Fig. S1 of the supporting information). In one of these structures, obtained by crystallization in the LCP (PDB entry 1ogv; Katona *et al.*, 2003 ▸), unidentified positive difference electron densities in the binding site of the ubi­quinone *Q*_B_ [Fig. S1(*a*)] and in the binding site of carotenoid (Fig. S1b) were observed. The same pattern was observed in the case of the RC structure obtained from crystallization in the LSP [Wadsten *et al.*, 2006 ▸; PDB entry 2gnu; Figs. S1(*c*) and S1(*d*)]. The electron densities in these crystal structures are very similar to what we have observed in our structures. Figs. S1(*b*) and S1(*d*) show our attempt to fill the carotenoid binding site in these structures with the monoolein molecule. The electron density found in the *Q*_B_ binding site can also be attributed to the monoolein molecule [Fig. S1(*a*)].

In previous work, a similar feature of the mesophase structure of the RC from purple bacterium *Blastochloris (Bl.) viridis*, which was crystallized in LSP, was observed (Wöhri *et al.*, 2009 ▸). This photosynthetic RC is similar to the RC of *Rba. sphaeroides*, except for the presence of the additional subunit C. The resulting structure revealed the presence of a non-native cofactor in the binding region of the secondary ubi­quinone *Q*_B_. The authors described this feature and fit in a molecule they consider to be monoolein but when viewed from the crystal structure is actually an MPG {[(Z)-octadec-9-enyl] (2*R*)-2,3-bis(oxidanyl)propano­ate} compound which is probably the result of a mistake in the HIC-Up database where the MPG molecule was described as a monoolein molecule (PDB entry 2wjm, Fig. S2). In this RC a 15-*cis*-1,2-di­hydro­neurosporene molecule is normally located in the carotenoid binding pocket. It is present in both *in meso* and *in surfo* crystal structures (Wöhri *et al.*, 2009 ▸; Li *et al.*, 2006 ▸). Thus, in the lipid mesophase crystal structure of the *Bl. viridis* photosynthetic RC no anomalies in the carotenoid binding region have been observed.

The *Rba. sphaeroides* RC is a prospective object for structural and functional studies of photosynthesis. Since the object is photoactive, it is highly suitable for time-resolved serial investigations of the photochemical charge separation with no need for additional modifications. However, since the sphero­idene and secondary ubi­quinone are important for the proper functioning of this pigment-protein complex, their absence may cause problems for studies of photosynthetic processes in crystals obtained by crystallization in lipid mesophases.

One way to solve the problem of substitution or loss of natural cofactors may be an increase in their concentration in the crystallization mixture, which may help to displace the alien components from protein binding sites. The possibility of this approach was demonstrated here using RC co-crystallization with sphero­idene. However, we could not succeed in restoring ubi­quinone in the *Q*_B_ site probably due to its relatively low affinity to this site (which is closely related to its function) in comparison with monoolein. Another possible way is to replace monoolein with another suitable matrix lipid that has a different structure. To date, a number of studies have already shown the fundamental potential of such approach (Li *et al.*, 2011 ▸; Höfer *et al.*, 2011 ▸; Salvati Manni *et al.*, 2015 ▸; Ishchenko, Peng *et al.*, 2017 ▸; Borshchevskiy *et al.*, 2010 ▸).

*Rba. sphaeroides* RC crystals without secondary ubi­quinone are still of interest to study the ultrafast (from femotoseconds to picoseconds) steps of electron transfer from the bacterio­chloro­phyll dimer to the primary ubi­quinone by time-resolved serial crystallography. The absence of ubi­quinone *Q*_B_ makes it difficult to study only the last stage of the primary electron transfer from *Q*_A_ to *Q*_B_. Crystals obtained in this work were successfully tested using silicon microchips for fixed-target sample delivery, both at synchrotron beamlines and at the EuXFEL SFX station, and the results will be described and published elsewhere.

## 4. Materials and methods   

### 4.1. Protein preparation and purification   

The recombinant strain of *Rba. sphaeroides* that produced wild-type reaction centers was cultivated using Huttner medium containing kanamycin (5 µg ml^−1^) and tetracyclin (1 µg ml^−1^) as described previously (Khatypov *et al.*, 2005 ▸). Reaction centers were isolated and purified using two-step ion-exchange chromatography as described in the work by Fufina *et al.* (2007 ▸).

After purification the RCs were solubilized in 20 m*M* Tris–HCl buffer (pH 8.0) containing 0.1% *N*,*N*-di­methyldo­decyl­amine-*N*-oxide (LDAO). Room-temperature absorption spectra of the RC were recorded with a Shimadzu UV1800 PC spectrophotometer (Japan). Sample purity *A*_280_/*A*_800_ was <1.4.

### 4.2. Crystallization   

For the crystallization we used 1-mono-oleyl-rac-glycerol (MO) of 99% purity and the 1,2,3-heptanetriol high melting point isomer (>98% purity) purchased from Sigma–Aldrich; Jeffamine M600 was purchased from Hampton Research and LDAO (>99% purity) was purchased from Sigma–Aldrich.

For crystallization experiments we used RC solutions with a protein concentration of 25–30 mg ml^−1^. The lipidic sponge phase was prepared by mixing lipidic cubic phase and buffer solution, as described in the work by Wadsten *et al.* (2006 ▸).

For RC crystallization, we used the previously described mesophase crystallization in the LCP (Katona *et al.*, 2003 ▸) and in the LSP (Wadsten *et al.*, 2006 ▸). For LSP crystallization trials we used the hanging-drop vapor-diffusion method and for the LCP trials we used microbatch crystallization. Diffraction data were collected at the ESRF in Grenoble and at PETRA III (DESY) in Hamburg. In addition, we carried out RC crystallization by vapor diffusion in a hanging drop with the addition of detergent under the conditions established in our earlier work (Gabdulkhakov *et al.*, 2013 ▸).

### 4.3. Dissolving crystals for spectroscopic analysis   

The crystals were dissolved in 200 µl 20 m*M* Tris–HCl buffer pH 8.0 containing 1% LDAO. Samples were then centrifuged for 2 min at 5000*g* to remove undissolved RC crystals. Optical absorption spectra of the isolated RCS were measured using a Shimadzu UV-1800 PC spectrophotometer (Japan): the path length was 10 mm. Sodium ascorbate was added at 1 m*M* final concentration in order to keep the primary donor in the reduced state.

### 4.4. Crystallization in Hamilton glass syringes   

For this technique we used 100 and 250 µl Hamilton gas-tight glass syringes. At first we mixed LSP with a protein solution (1:1 ratio to a final volume of about 80 µl) inside two connected 100 µl syringes until the mixture was homogeneous. Finally LSP/protein mix was transferred into one syringe. Another syringe was disconnected from the connector. A 250 µl syringe was filled with a salt counter solution (2 *M* tris­odium citrate). After disposing of the residual air in the syringe with salt solution, it was connected to the LSP-containing syringe. The LSP/protein mix was smoothly injected into the salt solution.

The syringe containing salt solution and the LSP/protein mixture was positioned horizontally allowing LSP to be spread in a thin layer. Incubation was carried out in a syringe wound with Parafilm in the dark at 16°C. RC crystals began to form on the second day, and reached their maximum size after 7 d. In this way, we were able to obtain a homogeneous mixture of crystals [Figs. 2 ▸(*a*) and 2 ▸(*b*)]. However, this approach only allowed us to obtain crystals that could diffract to a maximum resolution of 2.8 Å.

### 4.5. In-tip crystallization   

To increase the quality of the crystals, we established a new approach of mesophase crystallization. Instead of glass syringes, we used 1–10 µl plastic tips for automatic pipettes to incubate the crystallization mixture (Fig. S3). Positioned horizontally, they allow a vast area of the phase boundary to be saved, while, due to the cheapness and availability of the tips, we optimize crystallization conditions faster and more efficiently than upon crystallization in syringes. The scheme of this experiment is presented in Fig. S4. In-tip crystallization allowed titration of the salt solution concentration and control of its volume ratio to the LSP. Injection of salt solution and LSP/protein mix inside the tip was performed using Hamilton gas-tight syringes. To isolate the crystallization mixture from ambient air, the narrow end of the tip was first sealed, and after adding all the components of the mixture, the wide end of the tip was tightly wrapped in Parafilm.

We used tris­odium citrate with concentrations from 1 to 2 *M* as a salt counter solution. We selected crystallization conditions in which large crystals (about 70 µm) suitable for X-ray diffraction experiments were formed. Diffraction data were collected and the crystal structure was refined up to 2.1 Å. By applying different conditions larger quantities of smaller crystals (about 20 µm) were obtained [Figs. 2 ▸(*c*) and 2(*d*)]. These crystals were tested and they diffracted up to 1.8 Å resolution. Such crystals can be used for serial diffraction data collection. The maximum volume of LSP containing crystals we obtained from one tip was about 30 µl, which is less than the maximum volume from Hamilton syringes, but much larger than when using the hanging-drop method (about 1 µl of crystal-filled LSP per drop). To extract crystals from the tip, the tip was cut off and either the LSP was removed with another tip, or the tip was put on an automatic pipette and the mixture was squeezed out.

### 4.6. Preparation of sphero­idene and ubi­quinone   

Both sphero­idene and ubi­quinone were dissolved in suitable organic solvents. To dissolve sphero­idene we used acetone. The extraction method for this pigment is described in the work by Ashikhmin *et al.* (2017 ▸).

Ethyl acetate was chosen to dissolve ubi­quinone, as it is one of the most effective solvents for this substance (Zhao *et al.*, 2013 ▸). We dissolved ubi­quinone (Coenzyme Q10) powder (Sigma–Aldrich) in ethyl acetate until the solution was completely saturated.

### 4.7. X-ray diffraction analysis   

X-ray diffraction data were collected at the BL14.1 beamline at BESSY II (Berlin, Germany) at 100 K using a crystal grown under *in surfo* conditions.

Diffraction data from the LCP were collected on the ID23-1 beamline at the European Synchrotron Radiation Facility (ESRF), Grenoble, France (Nurizzo *et al.*, 2006 ▸), equipped with a PILATUS 6M detector. Data collection was controlled by the *MxCuBE* system (Gabadinho *et al.*, 2010 ▸) and the strategy was calculated by *BEST* (Bourenkov & Popov, 2010 ▸).

A dataset from the RC crystallized in LSP with carotenoid was collected at beamline P11 at PETRA III (DESY, Hamburg, Germany). All data were processed and scaled with the *XDS* package (Kabsch, 2010 ▸).

The structures were solved by molecular replacement using *Phaser* (McCoy *et al.*, 2007 ▸) and the structure of the photosynthetic RC from *Rba. sphaeroides* strain RV (PDB entry 3v3y; Vasilieva *et al.*, 2012 ▸) was used as a search model. The initial model was subjected to crystallographic refinement using *REFMAC5* (Murshudov *et al.*, 2011 ▸). Manual rebuilding of the model was carried out using *Coot* (Emsley *et al.*, 2010 ▸). Data and refinement statistics are summarized in Tables 1 ▸ and 2 ▸. The coordinates and structure factors have been deposited in the Protein Data Bank with the PDB entries 6z02 and 6z1j. Figures were prepared using *PyMOL* (DeLano, 2002 ▸).

## Supplementary Material

Supporting figures. DOI: 10.1107/S2052252520012142/cw5028sup1.pdf

PDB reference: Photosynthetic reaction center from *Rhodobacter sphaeroides* strain RV, *in surfo* crystallization, 6z02

PDB reference: LCP crystallization, 6z27

PDB reference: LSP co-crystallization with sphero­idene, 6z1j

## Figures and Tables

**Table 1:** Data collection and processing

	LCP crystallization	LSP co-crystallization with carotenoid	*In surfo* structure
Diffraction source	ESRF, beamline ID23-1	PETRA III, DESY beamline P11	BESSY, beamline BL14.1
Wavelength (Å)	0.8610	1.033	0.9184
Photon flux at sample [photons s^−1^ (100 mA)^−1^]	4 × 10^12^	8 × 10^12^	1.4 × 10^11^
Temperature (K)	100	100	100
Detector	Dectris PILATUS3 S 6M pixel	Dectris PILATUS3 S 6M pixel	Dectris PILATUS3 S 6M pixel
Crystal–detector distance (mm)	426	413	236
Rotation range per image (°)	0.05	0.1	0.1
Total rotation range (°)	150	150	180
Exposure time per image (s)	0.037	0.05	2
Space group	*P*4_2_2_1_2	*P*4_2_2_1_2	*P*3_1_21
*a*, *b*, *c* (Å)	99.9, 99.9, 234.1	99.9, 99.9, 238.5	139.9, 139.9, 185.4
α, β, γ (°)	90, 90, 90	90, 90, 90	90, 90, 120
Mosaicity (°)	0.05	0.053	0.14
Resolution range (Å)	50.0–2.07 (2.13–2.07)	50–2.1 (2.15–2.1)	50–2.1 (2.2–2.1)
Total No. of reflections	802655 (62473)	778321 (48871)	579056 (56011)
No. of unique reflections	72564 (5862)	71322 (5199)	121690 (15297)
Completeness (%)	99.9 (100.0)	99.9 (100.0)	99.5 (96.9)
Redundancy	11.06 (10.66)	10.91 (9.40)	4.75 (3.66)
〈*I*/σ(*I*)〉	15.94 (1.14)	9.61 (1.31)	12.52 (1.94)
*R*_r.i.m._	0.075 (184.6)	0.199 (133.2)	0.079 (68.8)
Overall *B* factor from Wilson plot (Å^2^)	52.20	35.59	36.1

**Table 2:** Structure solution and refinement

	LCP crystallization	LSP co-crystallization with carotenoid	*In surfo* structure
PDB entry	6z27	6z1j	6z02
Resolution range (Å)	48.86–2.10 (2.16–2.10)	46.07–2.10 (2.13–2.10)	43.2–2.1 (2.13–2.1)
Completeness (%)	100.0	100.0	99.6
σ cutoff [*F* > σ(*F*)]	1.36	1.36	1.99
No. of reflections (working set)	70033 (5613)	67749 (2680)	121683 (3 591)
No. of reflections (test set)	1737 (143)	3567 (142)	6085 (189)
Final *R*_cryst_	0.209 (0.3120)	0.188 (0.2932)	0.201 (0.357)
Final *R*_free_	0.236 (0.3420)	0.224 (0.3111)	0.226 (0.390)
Cruickshank DPI (Å)	0.18	0.16	0.12
No. of non-H atoms			
Protein	6409	6478	6471
Ion	0	6	18
Ligand	649	695	940
Water	161	185	444
Total	7219	7643	7873
R.m.s. deviations			
Bonds (Å)	0.008	0.008	0.007
Angles (°)	1.031	0.884	0.981
Average *B* factors (Å^2^)			
Protein	59.9	37.4	39.0
Ion	0	62.9	60.0
Ligand	61.18	40.4	46.3
Water	53.21	34.4	44.5
Ramachandran plot			
Most favored (%)	96.65	97.80	97.55
Allowed (%)	3.23	2.20	2.45
