# Supplementary material for: Novel approaches for the lipid sponge phase crystallization of the Rhodobacter sphaeroides photosynthetic reaction center
Source: IUCrJ. 2020 Oct 3;7(Pt 6):1084–91. doi: 10.1107/S2052252520012142 (PMC7642779; doi:10.1107/S2052252520012142)

IUCrJ

Volume 7 (2020)

Supporting information for article:

Novel approaches for lipid sponge phase crystallization of *Rhodobacter sphaeroides* photosynthetic reaction center

Georgii Selikhanov, Tatiana Fufina, Lyudmila Vasilieva, Christian Betzel and Azat Gabdulkhakov

Figure S1 Fragment of electron density map of the *Rba. sphaeroides* RC structure. (a) Q_B binding site (PDB entry code: 1OGV), pink molecule is a monoolein molecule fitted into an empty positive difference density. 2.35 Å, 2Fo-Fc electron density map is contoured at 1.8 σ. Fo-Fc is contoured at 2.5 σ; (b) carotenoid binding site (PDB entry code: 1OGV), pink molecule is a monoolein fitted into an empty positive difference density. 2.35 Å, 2Fo-Fc electron density map is contoured at 1.8 σ. Fo-Fc is contoured at 2.0 σ; (c) Q_B binding site (PDB entry code: 2GNU). 2.2 Å, 2Fo-Fc electron density map is contoured at 1.6 σ. Fo-Fc is contoured at 2.0 σ and -2.0 σ; (d) carotenoid binding site (PDB entry code: 2GNU), pink molecule is a monoolein molecule fitted into an empty positive difference density. 2.2 Å, 2Fo-Fc electron density map is contoured at 1.8 σ. Fo-Fc is contoured at 2.5 σ

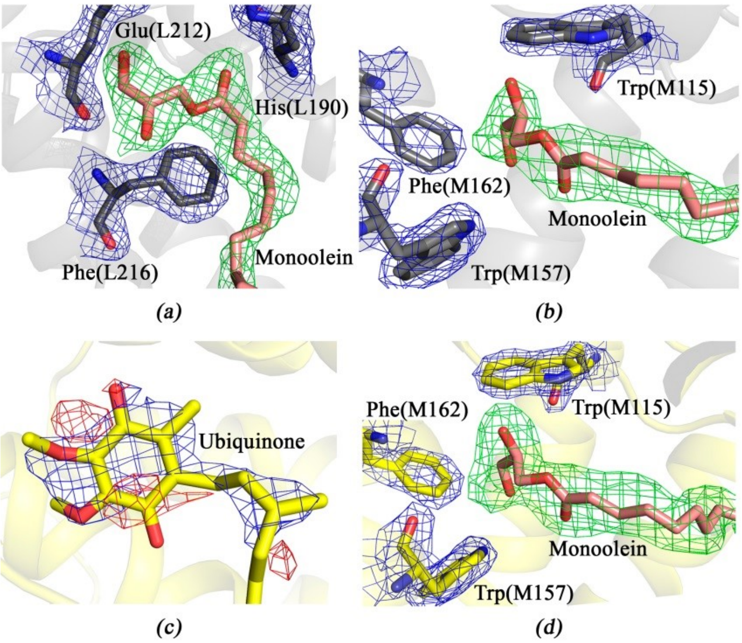

Figure S2 Fragment of electron density map of the *Bl. viridis* RC structure in the binding site of Q_B obtained with LSP crystallization technique (PDB entry code: 2WJM). 1.95 Å resolution, 1.6 σ

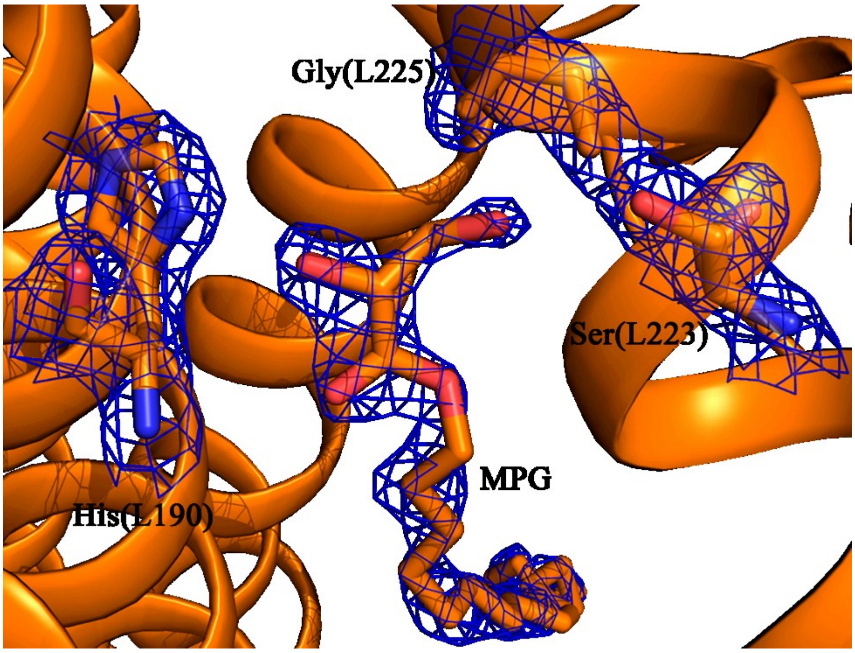

Figure S3 (a) Ultramicro Tip for Eppendorf Pipettors (Scientific Specialties Inc.), used for in-tips crystallization; (b) sealed tip; (c) sealed tip filled with salt solution and LSP with RC crystals. Base of the tip is wrapped in Parafilm

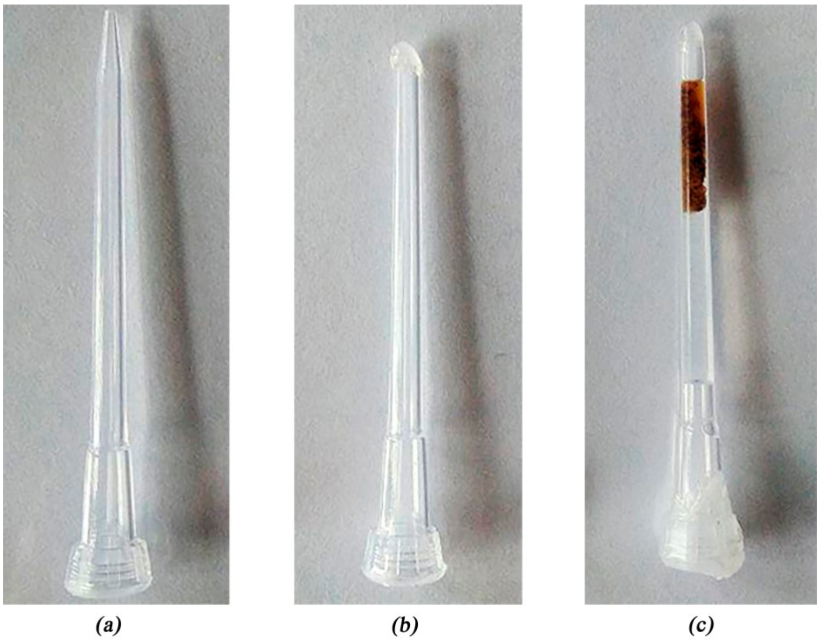

Figure S4 The scheme of in-tips crystallization: (1) empty tip; (2) empty tip is sealed on the tip; (3) tip is filled with salt solution; (4) LSP/protein mix is injected into the salt solution; (5) base of the tip is wrapped in Parafilm; (6) after the incubation crystals are formed in LSP

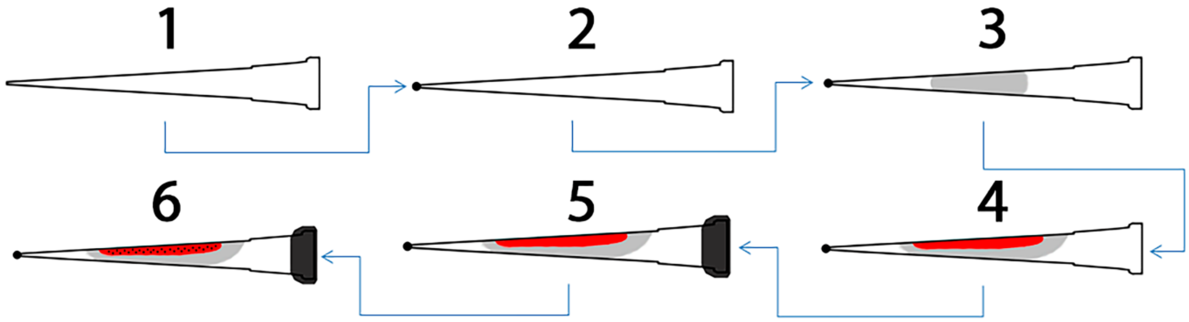

Supplement: Supplementary file 1 [file m-07-01084-sup1.pdf]
